# A Review of CPR Augmentation Devices

**DOI:** 10.7759/cureus.37350

**Published:** 2023-04-09

**Authors:** Moshe Bengio, Glenn Goodwin, Alexander Scumpia

**Affiliations:** 1 Emergency Medicine, Nova Southeastern University Dr. Kiran C. Patel College of Osteopathic Medicine, Davie, USA; 2 Emergency Medicine, Touro College of Osteopathic Medicine, Harlem, USA; 3 Emergency Medicine, Lakeside Medical Center, Belle Glade, USA

**Keywords:** emergency medicine, prehospital emergency care, emergency medical services, decompression, cardiopulmonary resuscitation, cardiac arrest

## Abstract

The study aims to assess cardiopulmonary resuscitation (CPR) outcomes in cardiac arrest patients when using CPR augmentation devices, such as the ZOLL ResQCPR system (Chelmsford, MA) or its components ResQPUMP and ResQPOD, which are manual active compression-decompression (ACD) device and impedance threshold device (ITD), respectively. The analysis included a Google Scholar-based literature review that took place between January 2015 and March 2023 and included recent publications with PubMed IDs or widely cited articles to assess the effectiveness of the ResQPUMP and ResQPOD or similar devices. This review also includes studies quoted by ZOLL, but those were not considered in our conclusion since the authors were employed by ZOLL. We found that in a study on human cadavers, the force of decompression increased the chest compliance of the chest wall by 30%-50% (p<0.05). Essentially, active compression-decompression improved the return of spontaneous circulation (ROSC) with meaningful neurologic outcomes by 50% in a blinded, randomized, and controlled human trial (n=1,653; p<0.02). The main study on the ResQPOD had a controversial human data pool with one randomized and controlled study arguing for no significant difference with or without the device (n=8,718; p=0.71). However, a post hoc analysis and the reorganization of the data by CPR quality demonstrated significance (n decreased to 2,799, reported in odds ratio without specific p-values). In conclusion to the limited number of studies presented, any manual ACD device is a great alternative to standard cardiopulmonary resuscitation regarding survivability with good neurologic function and should be utilized in prehospital emergency medical services and hospital emergency departments. ITDs are still controversial but promising with more future data.

## Introduction and background

There has been a marked increase in out-of-hospital cardiac arrest (OHCA) encounters over the last decade, increasing from approximately 326,000 in 2012 to 356,000 in 2022 [1-3]. Of those in 2020, emergency medical services (EMS) performed emergency resuscitative measures, including cardiopulmonary resuscitation (CPR) and defibrillation, on 127,000. Nine percent of those patients survived to discharge and 7.0% with good neurologic outcomes [3]. The 2020 survival-to-discharge percentage decreased from 2019 by 1.6%, likely due to COVID-19 [3].

Historically, the American Heart Association (AHA) introduced cardiopulmonary resuscitation (CPR) in the late 1950s. Aufderheide et al. published the concept of "less is more" when they discovered the correlation between higher ventilation rates and worsened survival in cardiac arrest trials [4]. The actual ventilation rates worsened outcomes via two mechanisms: obstructing compressions and increased intrathoracic pressures (ITP), which have irrefutably been found to decrease preload and coronary perfusion pressures (CPP) [4]. Then, in the 1990s, Paradis et al.'s study demonstrated the link between CPR, CPP, and return of spontaneous circulation (ROSC), virtually changing EMS protocols overnight [5]. Many landmark studies began focusing on various aspects of CPR optimization, such as compression depth, rate, and minimization of interruptions [6-9]. Ten years later, the resuscitation landscape was revolutionized again, pertaining to the core concepts of compression release velocity during full compression recoil or active decompression. This led to the advent of active compression-decompression (ACD) and impedance threshold devices (ITDs).

Physiology

Full recoil occurs when the ribs return to their original position dictated by the compliance of the chest wall [10]. The full recoil effect, which is normally thought of as a passive process, can be inhibited by user dependance (i.e., incomplete recoil or incomplete compression) or fractured ribs, both of which minimize the distance, a factor of pressure [11]. However, pressure is also a function of velocity. Therefore, actively stumping recoil velocity also inhibits the return to optimal intrathoracic pressure (ITP). For example, a provider may become fatigued and does not completely remove his or her body weight from the patient prior to recoil; even if full recoil is eventually achieved, the slower speed of the recoil mitigates the ideal intrathoracic pressure for significant venous return. Accordingly, a positive neurologic outcome is a better measure of success than achieving ROSC since hypoxic changes rapidly effect the brain more than other organs due to deficient regenerative means [12].

Resuscitation assist devices

Over the years, many products have been introduced to maximize the multiple components of CPR for cardiac arrest to improve patient outcomes [13]. One study found that doubling recoil velocity quadruples survival rate and significantly improves neurologic outcomes [14]. Additional studies have corroborated those findings in both animal and human studies [15,16]. These studies and others have led to the development of many active compression-decompression (ACD) devices. One of the more ubiquitous devices is the Lund University Cardiopulmonary Assist System (LUCAS) device, which utilizes an automatic suction cup on a piston-based chest compression apparatus. The suction cup pulls the compressed chest back to the decompressed position, actively recoiling the thorax, increasing preload, particularly the preload velocity [17]. The advantage is twofold: it not only acts as an ACD but also performs compressions automatically, freeing up healthcare personnel to perform other tasks [17]. Unfortunately, the LUCAS device can be cost-prohibitive. An alternative, nonmechanical, affordable ACD option is the ResQPUMP. The cost of a ResQPUMP is like that of an automated external defibrillator (AED), which is commonly available in public spaces.

**Figure 1:**
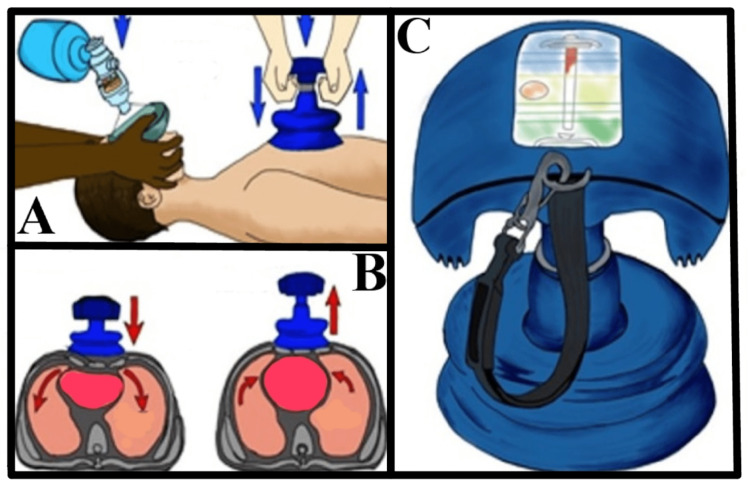
ResQCPR system illustration This figure was modified with permission from Goodwin et al. [19] who recreated the depiction of the ResQCPR system that was included in Lobodzinsk's review [18] of the ResQ trial. (A) The ResQPOD on the left is connected to the bag valve mask (BVM), and the ResQPUMP on the right is involved in chest compressions. (B) The left-side image shows the compression phase with blood pumped from the heart to the periphery. On the right, the chest is actively decompressed allowing for better venous return. (C) Another view of the ResQPUMP and its monitor, which acts as a guide for optimal compression depth, rate, and decompression force

In addition to ACD devices are impedance threshold devices (ITDs). ITDs attach to the patient's endotracheal tube. The attachment acts as a one-way valve, allowing for exhalation but preventing air entry during recoil, theoretically maximizing negative ITP. See Figure 1 for an illustration on the ResQCPR system (ZOLL, Chelmsford, MA) [18,19]. Several ACD devices and ITDs exist, but this manuscript will highlight the ZOLL ResQCPR system as the representative sample, which is composed of the ResQPUMP (ACD) and the ResQPOD (ITD). The ResQPUMP and its counterpart, ResQPOD, make up the ZOLL ResQCPR system and can be used together or separately. The ResQPUMP is a manual nonautomated device that pulls the chest upward during the recoil portion of CPR via suction cup, effectively accomplishing excellent recoil velocity or active decompression. The ResQPOD is an ITD that aids the ResQPUMP. The company claims that in two separate studies, the one-year survival rate was increased by 49% [20]. The focus of this report will address the limited but convincing independent studies available on the ZOLL ResQCPR system and its ability to improve survival and neurologic function.

## Review

Methods

This paper follows up on Goodwin et al.'s 2021 review published in the International Journal of Innovative Research in Medical Science and clarifies its recorded studies or supplements its findings with newer or alternative studies [19]. The authors of this manuscript conducted a literature review between January 2015 and March 2023 on Google Scholar. Initially, the search was limited to English resources with PubMed IDs that were recently published pertaining to the ResQCPR system, ResQPUMP, or ResQPOD; however, due to the scarcity of research on these items in the past years, our search was further expanded to include active compression-decompression (ACD) or impedance threshold device (ITD). Referenced papers within those articles were also selectively included based on relevance and if widely referenced. Therefore, there are a few articles included from as far back as 2002. Other studies are included in lesser detail but with a focus on references to solidify or challenge the research with current research. Lastly, studies sponsored by, or an author affiliated with, ZOLL were included for reference but not in the study's conclusive findings.

Preface

When breaking down the components of successful CPR, it will become evident that CPR augmentation devices, such as the ResQCPR system, or, at the very least, ACD devices, such as the ResQPUMP, are superior to manual CPR. The main components discussed here are compression, active decompression, compliance, ROSC, neurologic outcomes, intrathoracic pressures, venous return, and contrast to the LUCAS device.

Chest compliance in active compression-decompression

**Figure 2:**
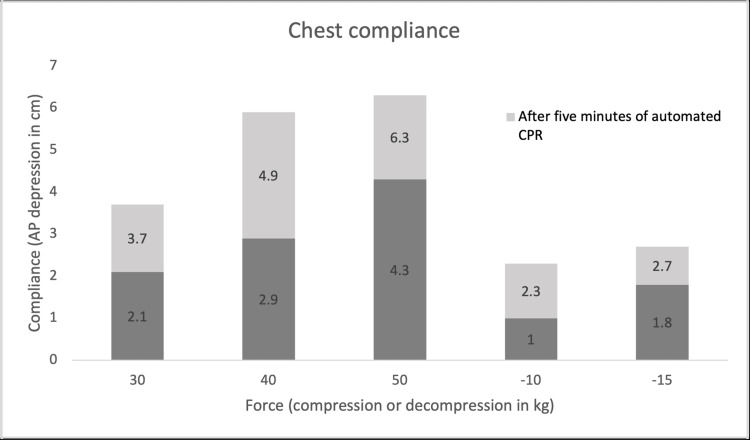
Chest compliance This graph replicates the data from Segal et al., establishing an increase in compliance after five minutes of automated CPR at fixed forces composed of compressive and active decompressive components [22] AP, anterior-posterior; CPR, cardiopulmonary resuscitation

Compliance is measurable via the capacity to decrease the anterior-posterior (AP) chest height during compression [21]. Segal et al. found that in non-frozen, very recently deceased cadavers, the ACD increased the compliance after compressive and decompressive components, which were derived using lateral chest X-rays, as demonstrated in Figure 2 [22]. Segal et al. also correlated this increased compliance with greater CPR effectiveness, and these findings were further substantiated by multiple subsequent studies [14-16]. The findings of Segal et al. are illustrated in Figure 2. It must be emphasized that the Segal et al.'s study utilized the LUCAS device, not the manual ACD device such as the ResQPUMP. However, it is the active and forceful recoil, which is shared by the two devices, that is the driving force of increased compliance [23,24].

Neurologic outcomes versus ROSC

**Figure 3:**
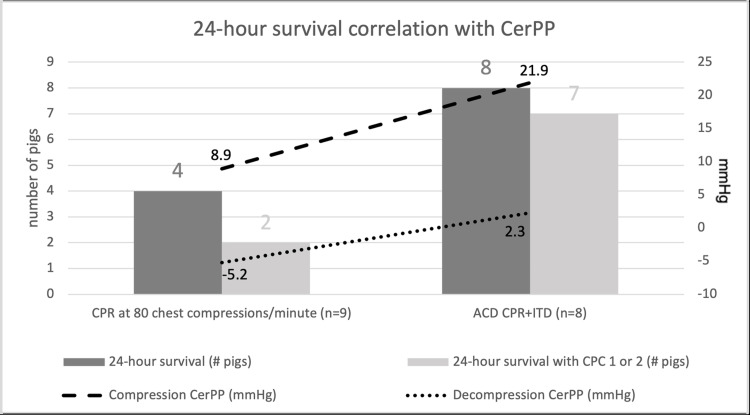
Twenty-four-hour survival graph correlation with cerebral perfusion pressure (CerPP) Twenty-four-hour survival represents ROSC only, and 24-hour survival with cerebral performance score (CPC) 1 or 2 represents neurologic outcome. Cerebral performance score (CPC) of 1 or 2 indicates a positive neurologic outcome on a 1 (normal) to 5 (death) point scale. Metzger et al.'s data reflected here shows significant neurologic survival in the active compression-decompression CPR+impedance threshold device (ACD CPR+ITD) group at 88% versus the 80 cc/minute CPR without decompressive or ITD focus at 22% (p<0.02). ROSC alone was 100% in the ACD CPR+ITD versus 44% in the compression-only group (p<0.05). It is evident that neurologic survival is correlated with mean cerebral perfusion pressure (CerPP) calculated here after 10 minutes of resuscitation during compression (p<0.00001) and decompression (p<0.01) phases [25] CPR, cardiopulmonary resuscitation; ROSC, return of spontaneous circulation

Metzger et al. proved in a study on pigs that the addition of active decompression with ResQCPR system including the ResQPUMP and the ResQPOD increased neurologic outcomes at 24 hours. Being that the study by Metzger et al. was the impetus for the ResQCPR system, ResQPUMP and ResQPOD are described without their brand name as active compression-decompression (ACD) CPR and impedance threshold device (ITD), respectively. Neurologic outcome at 24 hours was evaluated by a higher grading of a cerebral performance score versus low scoring but the achievement of ROSC as shown in Figure 3. In a parallel cohort, the study group with ACD CPR+ITD was shown to have significantly increased cerebral perfusion pressure, likely the reason for the better neurologic outcome (Figure 3 lines) [25]. Kovacs et al. verified that Metzger et al.'s study findings are translatable to human subjects. This study reviewed outside of hospital cardiac arrests retrospectively from a five-year period using a ZOLL defibrillator with accelerometer-based technology. The researchers were able to correlate the chest compression release velocity (CCRV), which is essentially active decompression, with increased survival and neurologic outcomes [14]. It is worth noting that Kovacs et al. did not use an ITD device, the significance of which will be elaborated in the forthcoming section.

**Figure 4:**
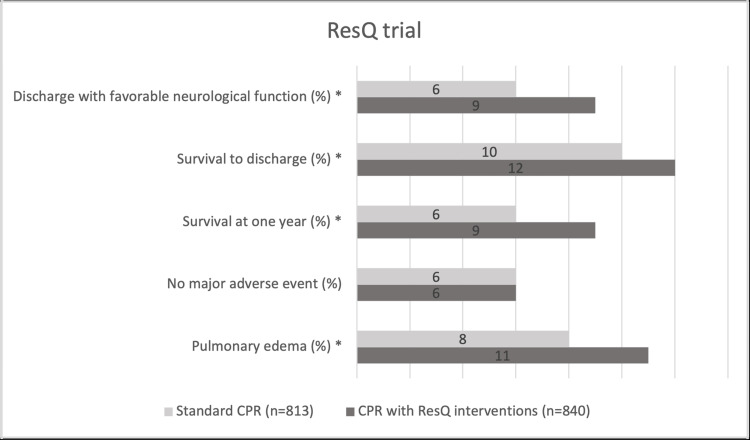
The ResQ trial The statistically significant results of Aufderheide et al.'s study exhibited in this graph reveal the superiority of ResQCPR versus standard CPR. The primary outcome of neurologic function is 50% better in the ResQCPR group defined by a Rankin score of ≤3 (p<0.02). A secondary outcome of survival at one year was also significantly greater in the ResQCPR group (p<0.05). Pulmonary edema was interestingly but perhaps not surprisingly more likely in the ResQCPR group given the mechanism of the device (p<0.02) [26] *Statistically significant CPR: cardiopulmonary resuscitation

The most promising neurologic outcome study was the ResQ trial by Aufderheide et al. The ResQ trial was a large, blinded, randomized, controlled trial between 46 EMS agencies in rural, suburban, and urban areas that included 1,653 non-traumatic prehospital cardiac arrest patients. They found that those in the ResQCPR group had 50% better neurologic function at discharge and survival at one year (Figure 4) [26]. Some of the researchers from the ResQ trial reanalyzed the data using different exclusion criteria (including more non-traumatic cardiac arrest patients) and still produced equivalent findings [27]. A limitation of both the Metzger et al. and ResQ trial studies is that the researchers did not differentiate ResQPUMP versus ResQPOD in isolation from one another. Both devices were used, possibly potentiating their respective results. ZOLL claims that the ResQCPR system must be used together to have meaningful improvement of outcomes. Later studies in animals and humans reconfirmed that active decompression, the mechanism or ResQPUMP, increases brain tissue oxygenation compared to passive decompression, leading researchers to question the practical utility of the ResQPOD [15,16,18].

ResQPOD

The one-way valve of the ResQPOD theoretically optimizes preload and increases venous return [20]. The ResQPOD manufacturers quote Metzger et al. and another similar study in pediatric populations as evidence of its effectiveness for cardiac arrest survival with significant neurologic outcomes [25,28]. As discussed in the previous section, Metzger et al. performed the study using the ACD and ITD combination [25]. These two devices were not studied individually. Kovacs et al. strongly suggest that the significance of Metzger et al.'s study is due to the ACD and not the ITD [14,25].

Other studies continue to doubt the effectiveness of the ITD. One study by Johnson et al. (summarized in Table 1) assessed whether the same theoretical optimization could be seen regarding systemic medication distribution. They conducted a randomized controlled pig trial. Cardiac arrest was induced by using IV potassium, and then, CPR was initiated with a LUCAS device with cycles of 30 compressions and two ventilations. Half of the porcine subjects were ventilated with the ResQPOD in place, and the other just had a standard endotracheal tube. Epinephrine was administered every four cycles in each group, with blood samples taken at baseline and fixed time intervals for all porcine subjects. The researchers argued that in the setting of increased venous return, which is when the ResQPOD is active, the heart would be able to take up more epinephrine leading to decreased systemic concentrations of epinephrine (Cmax). Consequently, the epinephrine will also take longer to reach peripheral circulation (Tmax) [29]. While a difference between the groups was seen, the results were not statistically significant, as demonstrated in Table 1 [30].

**Table 1:** Resulting epinephrine in standard intubation versus ResQPOD incorporation The maximum concentration of epinephrine in plasma is Cmax in nanograms/milliliters. The Tmax is the time in minutes to the maximum concentration of plasma epinephrine. MANOVA means the multivariate analysis of variances between the two groups and variables. Johnson et al. found no significant difference with p>0.05 [30]

ResQPOD utilized	Variable factor	Mean	Standard deviation	MANOVA
No (n=6)	Cmax (ng/mL)	471.53	349.71	p=0.276
Yes (n=6)		219.34	110.59	
No (n=6)	Tmax (minutes)	3.42	1.11	
Yes (n=6)		4.75	1.54	

The ROC PRIMED trial was another attempt to validate the ITD device. The trial was a randomized controlled study among EMS-managed cardiac arrests including nearly 8,700 human subjects. The primary endpoint was survival to discharge with significant neurologic function based on the Rankin scale. EMS in both arms of the trial was trained on the proper use of the ITD with the treatment arm using an actual ITD and the control arm a sham device. The results were again nil, with p-value demonstrating a insignificant difference in endpoint (p=0.71) [31].

**Figure 5:**
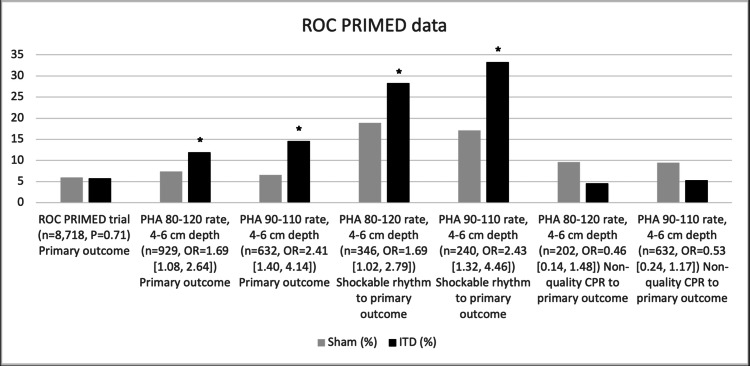
ROC PRIMED data This figure compares the original ROC PRIMED study with the post hoc analysis (PHA) follow-up study on the same date. The original study compared ITD to sham without investigating CPR integrity. The PHA study divides the data based on CPR quality. The primary outcome was survivability to discharge with good neurologic outcome based on the Rankin criteria. The secondary outcome was subjects with shockable rhythms and ultimately survivability to discharge with good neurologic function. The bars represent a percentage of either the sham ITD or the active ITD that met the measured outcome. The odds ratio (OR) was used as a measure of significance in the PHA study and is included with a 95% confidence interval range in brackets [31,32] *Statistically significant n, number of subjects included in the study; ITD, impedance threshold device; CPR, cardiopulmonary resuscitation

However, in a post hoc analysis of the data from the ROC PRIMED trial, Sugiyama et al. argued that there was a significant benefit of the ITD to the primary endpoint. Sugiyama et al. reorganized the data into quality CPR and non-quality CPR. All unwitnessed out-of-hospital cardiac arrests and rescues that did not have recorded compression rate and depth were excluded. When the compression rates of 80-120 and the depths of 4-6 cm were achieved, survivability with significant neurologic function was significantly better in the ITD group versus the sham. Sugiyama et al. further stratified the data into 90-110 compressions and 4-6 cm depth. In doing so, survivability with significant neurologic function was further improved in the ITD group versus the sham. When the quality of the CPR was neglected from the analysis, the difference between the two arms was insignificant. These findings seem to demonstrate a synergistic and potentiating effect of ITDs and quality CPR [32]. See Figure 5 for a comparison of the results from the original ROC PRIMED trial to the post hoc analysis.

Due to the conflicting studies and inconclusiveness on the effect of ITDs, the American Heart Association (AHA) recommends against the use of ITDs in conventional CPR unless combined with ACD in the setting of trained personnel in 2020 [1].

LUCAS device

Another ACD, the LUCAS device, has seen a huge increase in utilization for its added benefit of automatization. This comes at a cost, however, when one considers the injuries associated with automated ACD devices, including but not limited to sternal and rib fractures with associated hemorrhaging or lung injuries, liver lacerations, and aortic rupture [33,34]. Additionally, there has not been strong evidence demonstrating a mortality benefit of automated ACD devices, with a meta-analysis on multiple retrospective studies showing no benefit versus a single randomized controlled study with a mortality benefit [35,36]. We would postulate that manual ACD devices would be similar to manual CPR in regard to associated injuries; however, the authors would like to see future studies comparing adverse effects of the automated ACD devices to manual ACD devices.

## Conclusions

The current AHA guidelines permit the simultaneous utilization of both adjuncts of the ResQCPR apparatus. However, there is more evidence, in a limited research area, as established in this article supporting the practical benefit of ACD devices, such as the ResQPUMP, than ITDs, such as the ResQPOD. This benefit is particularly significant with regard to survival with good neurologic outcome. Given the similarities between the manual and automated ACD devices, with the exception of automatization, the authors wonder if manual devices, such as ResQPUMP, albeit less known, are the better of the two devices given the manual devices' potential for less adverse effects. Moving forward, we hope to see more utilization of manual ACD devices in both prehospital and hospital settings.
